# Reciprocal Hosts' Responses to Powdery Mildew Isolates Originating from Domesticated Wheats and Their Wild Progenitor

**DOI:** 10.3389/fpls.2018.00075

**Published:** 2018-02-23

**Authors:** Roi Ben-David, Amos Dinoor, Zvi Peleg, Tzion Fahima

**Affiliations:** ^1^Department of Vegetables and Field Crops, Institute of Plant Sciences, Agricultural Research Organization-Volcani Center, Rishon LeZion, Israel; ^2^Department of Plant Pathology and Microbiology, The Robert H. Smith Faculty of Agriculture, Food and Environment, Hebrew University of Jerusalem, Rehovot, Israel; ^3^The Robert H. Smith Faculty of Agriculture, Food and Environment, The Robert H. Smith Institute of Plant Sciences and Genetics in Agriculture, Hebrew University of Jerusalem, Rehovot, Israel; ^4^Department of Evolutionary and Environmental Biology, Faculty of Natural Sciences, Institute of Evolution, University of Haifa, Haifa, Israel

**Keywords:** wheat domestication, *Blumeria graminis tritici* (*Bgt*), resistance, wild emmer wheat, powdery mildew

## Abstract

The biotroph wheat powdery mildew, *Blumeria graminis* (DC.) E.O. Speer, f. sp. *tritici* Em. Marchal (*Bgt*), has undergone long and dynamic co-evolution with its hosts. In the last 10,000 years, processes involved in plant evolution under domestication, altered host-population structure. Recently both virulence and genomic profiling separated *Bgt* into two groups based on their origin from domestic host and from wild emmer wheat. While most studies focused on the *Bgt* pathogen, there is significant knowledge gaps in the role of wheat host diversity in this specification. This study aimed to fill this gap by exploring qualitatively and also quantitatively the disease response of diverse host panel to powdery mildew [105 domesticated wheat genotypes (*Triticum turgidum* ssp. *dicoccum, T. turgidum* ssp. *durum*, and *T. aestivum*) and 241 accessions of its direct progenitor, wild emmer wheat (*T. turgidum* ssp. *dicoccoides*)]. A set of eight *Bgt* isolates, originally collected from domesticated and wild wheat was used for screening this wheat collection. The isolates from domesticated wheat elicited susceptible to moderate plant responses on domesticated wheat lines and high resistance on wild genotypes (51.7% of the tested lines were resistant). Isolates from wild emmer elicited reciprocal disease responses: high resistance of domesticated germplasm and high susceptibility of the wild material (their original host). Analysis of variance of the quantitative phenotypic responses showed a significant Isolates × Host species interaction [*P*(F) < 0.0001] and further supported these findings. Furthermore, analysis of the range of disease severity values showed that when the group of host genotypes was inoculated with *Bgt* isolate from the reciprocal host, coefficient of variation was significantly higher than when inoculated with its own isolates. This trend was attributed to the role of major resistance genes in the latter scenario (high proportion of complete resistance). By testing the association between disease severity and geographical distance from the source of inoculum, we have found higher susceptibility in wild emmer close to the source. Both qualitative and quantitative assays showed a reciprocal resistance pattern in the wheat host and are well aligned with the recent findings of significant differentiation into wild-emmer and domesticated-wheat populations in the pathogen.

## Introduction

Genetic patterns of host susceptibility and pathogen virulence variation are essential underlying factors influencing disease epidemiology. Moreover, the distribution of this variation in space and time may shed light on the co-evolutionary selection mode between pathogens and plants. Relatively little empirical data on how ecological and evolutionary processes interact to influence the generation and maintenance of spatial and temporal variation in natural host–pathogen interactions has been collected (Tack et al., 2012). Moreover, studying the dynamics of pathogen and host at the center of origin where both natural and domesticated habitats co-exist sympatricaly could be extremely valuable.

Wild emmer wheat [*T. turgidum* ssp. *dicoccoides* (Körn.) Thell.] is the allo-tetraploid (2n = 4x = 28, BBAA) progenitor of both the tetraploid durum wheat [*T. turgidum* ssp. *durum* (Desf.) MacKey] and the hexaploid (2n = 6x = 42; BBAADD) bread wheat (*T. aestivum* L.) (Figure S1; Feldman, 2001). Native stands of wild emmer are distributed throughout the Near-Eastern “Fertile Crescent,” in the transition zone between the Mediterranean and the steppe phytogeographic provinces (Harlan and Zohary, 1966). In Israel and surrounding regions, this species thrives across a wide ecological amplitude, in diverse primary and, to a limited extent, secondary habitats (Kimber and Feldman, 1987). In some geographical regions, natural habitats of wild emmer are distributed distributed sympatrically with agricultural wheat fields.

Powdery mildew caused by the parasitic fungus *Blumeria graminis* (DC.) E.O. Speer, f. sp. *tritici* Em. Marchal (designated *Bgt* below), is one of the most devastating diseases of wheat, causing yield losses of up to 34% (Johnson et al., 1979). The life cycle of powdery mildew includes sexual (between seasons) and asexual (within season) stages, a strategy that combines the benefits of both new allelic combinations and often-effective off-season survival mechanisms (through sexual reproduction) with the advantages of rapidly multiplying individual clonal lines (through asexual reproduction) that are particularly adapted to specific host habitats (McDonald and Linde, 2002). Since powdery mildew propagates very efficiently on wild plants it forms a huge reservoir of parasites deeply rooted in wild host populations—a reservoir that, occasionally or constantly, could serve as a source of inoculums to initiate epidemics in the fields (Dinoor, 1974). Bawden (1957) suggested that in natural habitats, where the hosts are genetically heterogeneous “each plant is a selective ecological niche favoring only a few parasites.” In the long term, the genetically diverse mixture of wild hosts, in contrast to the genetically uniform cultivated fields, enables recombination and development of differing pathotypes, and can influence the pathogenic profile of the inoculums (Dinoor, 1974).

An *ex situ* collection of *Bgt* isolates (the “Eshed–Dinoor mildew collection,” Ben-David et al., 2014), has been established and maintained for more than two decades. Availability of both host and pathogen *ex situ* genetic collections enables us to use phenotypic and genotypic assays to examine issues of host/parasite co-evolution. Recent investigation of this *Bgt* collection has shown that based on host, it is significantly differentiated into wild-emmer and domesticated-wheat populations (Ben-David et al., 2016; Menardo et al., 2016). However, the results did not support the existence of a separate *B. graminis* f. sp. *dicocci* (Ben-David et al., 2016). In the present study, we examined a diverse set of wild and domesticated wheat lines with respect to their responses to inoculation with Israeli *Bgt* isolates collected from both wild and domesticated hosts.

We hypothesize that disease responses of diverse host span will be in accordance to this *Bgt* differentiation at the center of origin and that quantitative characterization will improve our understanding of Wheat-*Bgt* co-evolution. A set of eight distinct *Bgt* isolates were selected for inoculation of diverse host panel which includes tetraploid and hexaploid domesticated wheat, as well as their wild emmer progenitor. The specific aims were to: (i) analyze the phenotypic reactions of domesticated and wild wheat germplasm to inoculation with *Bgt* isolates, using both qualitative and quantitative scales; (ii) characterize the virulence and aggressiveness of *Bgt* isolates originating from domesticated and wild hosts; and (iii) assess the association between disease severity and geographic distance between sources of pathogen isolates and wild host wheat genotypes. Our results highlight the evolutionary dynamics between wheat and its pathogen *B. graminis* at the center of origin, where wild and domesticated wheats grow in intimate mixtures. Thus, they shed light on the co-evolutionary process underlying the sympatric distribution of domesticated wheat and its wild progenitor across the Fertile Crescent.

## Materials and methods

### Plant and pathogen material

A collection of 63 domesticated wheat genotypes, comprised of 16 bread wheats, 32 durum wheats, and 15 emmer wheats, together with 54 wild emmer accessions, was tested by means of detached-leaf assays at the seedling stage, for quantitative and qualitative responses to infection with powdery mildew (Table S1, Figure S2). These plant accessions comprise representative wheat collection of various habitats that encompass a wide eco-geographical range: 1–69.3°E; 8–52.5°N (Figure S3). The accessions originated from 18 different countries, the highest representations being from Israel (*n* = 25), Turkey (*n* = 14), Iran (*n* = 5), and Ethiopia (*n* = 5). The collection sites were not randomly distributed throughout the geographical area; the most numerous representations were comprised of wheat lines from the western arc of the Fertile Crescent (Figure S3). Hierarchical clustering (Ward, 1963) was used to identify discrete groups of sites, based on detailed eco-geographic profiles. Sites were selected to represent the six main clusters, which represent the maximum eco-geographic variance of the collection (Table S1). Hierarchical clustering was applied by using SPSS V21.0.0 (SPSS, 2004).

In addition, 42 wheat cultivars and 187 accessions of wild emmer were included in the qualitative phenotypic test. A set of differential wheat accessions carrying known Powdery mildew resistance genes (*Pm*) were obtained from the National Small Grain Collection (Aberdeen, Idaho, USA) and from Prof. F.J. Zeller (Technische Universität Munich, Institut für Pflanzenbau und Pflanzenzüchtung, Germany) (Table S2). This set of differential *Pm* wheat accessions is a common tool to characterize powdery mildew isolates based on virulence or avirulence against a specific *Pm* gene (e.g., Ben-David et al., 2016). All assays were performed on host plants at seedling stage. Pre-inoculation, plants were growing under controlled conditions, protected from pests and other pathogens.

*Bgt* isolates were collected from various wheat species (wild and domesticated) in various habitats across Israel (see Table 1 in Ben-David et al., 2010, Table S3). A single isolate—*Bgt*#GH—was collected from the cultivar Chinese Spring (designated CS below) in the greenhouse. Information regarding the origin of the isolates (excluding *Bgt*#GH) is presented in Table S2. In the present study, we have included six isolates for the quantitative assay: two isolates collected from durum wheat (*Bgt*#15 and *Bgt*#97), one isolate collected from bread wheat (*Bgt*#70) and three isolates collected from wild emmer (*Bgt*#58, *Bgt*#63, and *Bgt*#66). These six selected isolates represent both the virulence and genetic diversity of the Eshed—Dinoor mildew collection (Ben-David et al., 2016). In addition, two isolates collected from bread wheat (*Bgt*#101 and *Bgt*#GH) were included only in the qualitative phenotypic assay.

### Qualitative phenotypic assay

Conditions of incubation, inoculation, and disease assessment were according to Hsam and Zeller (2002) and Ben-David et al. (2010), with some modifications: The tests for mildew resistance were conducted on 10- to 14-days-old primary leaf segments maintained on agar at 6 g/l supplemented with benzimidazole at 50 mg/l in polystyrene boxes. Each box of leaf segments included genotypes from the wheat collection and susceptible genotypes [e.g., bread wheat cv. CS (for *Bgt*#15, *Bgt*#70, *Bgt*#97, *Bgt*#101, and *Bgt*#GH), durum wheat cv. Inbar (for *Bgt*#58), and wild emmer accession Israel-A, (PI-481521, USDA-ARS Cereal Crops Research Unit, Fargo, ND, USA) (for *Bgt*#66 and *Bgt*#63)] as controls. Each wheat genotype was represented by three replicates (a column of three leaf segments). Spore and germ-tube densities and germination rates were calculated for each box (recorded 24 h post inoculation, six random measurements per box) and were related to the average disease index of the control lines that were present in each box, in order to exclude a possible correlation between initial inoculum condition and final infection rate. No correlation was found between the initial spore and germ-tube densities and disease severity, and no correlation between germination rate and levels of disease response was detected.

Results of the powdery mildew tests were scored about 12 days after inoculation (dpi) and once again 2–5 days later. The infection types (IT) of powdery mildew were recorded on the basis of symptoms on a scale of 0–4, with 0 representing no visible symptoms and 4 fully infected leaves (Mains and Dietz, 1930). The IT was divided into three main categories: 0–2, 2–3, and 3–4 for resistant (R), moderate (M), and susceptible (S) reactions, respectively. To examine the association between frequency data of the three categories and host genetic source (wild/domesticated) a χ^2^ test for discrete variables was applied.

### Quantitative phenotypic assay

Conditions of incubation, inoculation and disease assessment were as described above. Quantitative assays were conducted on three separate dates, on each of which inoculation was performed with two different isolates: [Experiment I (*Bgt*#15 and *Bgt*#66); Experiment II (*Bgt*#70 and *Bgt*#58); and Experiment III (*Bgt*#97and *Bgt*#63)]. In each experiment, the leaf segments were scored quantitatively by counting the exact number of mildew pustules on each leaf segment 12 dpi. Area of leaf segments was measured by implementing the ImageJ software using the digital “Measure” function (http://rsb.info.nih.gov/ij/) and disease severity was calculated (No. of mildew pustules per Cm^2^).

### Statistical analyses

All statistical analyses were performed with the JMP statistical package (SAS Institute, Cary, NC, USA). ANOVA was applied to assess the effects of *Bgt* isolate and the source host, i.e., the wheat species from which mildew was sampled, on disease severity. An unbalanced nested factorial model was employed for all three experiments (see Supplementary File for model equation).

## Results

### Phenotypic tests with *Pm* differential lines

The results of the phenotypic assays with seven *Bgt* isolates on a set of 18 differential wheat lines, that carry different *Pm* genes or gene combinations, are presented in Table S2. The *Bgt* isolates interacted differentially with the *Pm* lines, each eliciting a specific reaction profile representing the differing specificities of the seven tested *Bgt* isolates. Isolate *Bgt*#66, collected from wild emmer was avirulent on all 17 differential *Pm* lines. *Bgt*#58 (like *Bgt*#66 collected from the same wild population at Amiad site, Eastern Galilee) and *Bgt*#63 were more virulent, overcoming, respectively, eight and seven of the *Pm* resistance genes (Table S2). The four *Bgt* isolates which originated from domesticated hosts were highly virulent on the differential set of lines, overcoming most of the represented *Pm* resistance genes.

### Qualitative phenotypic assay

**Figure 1:**
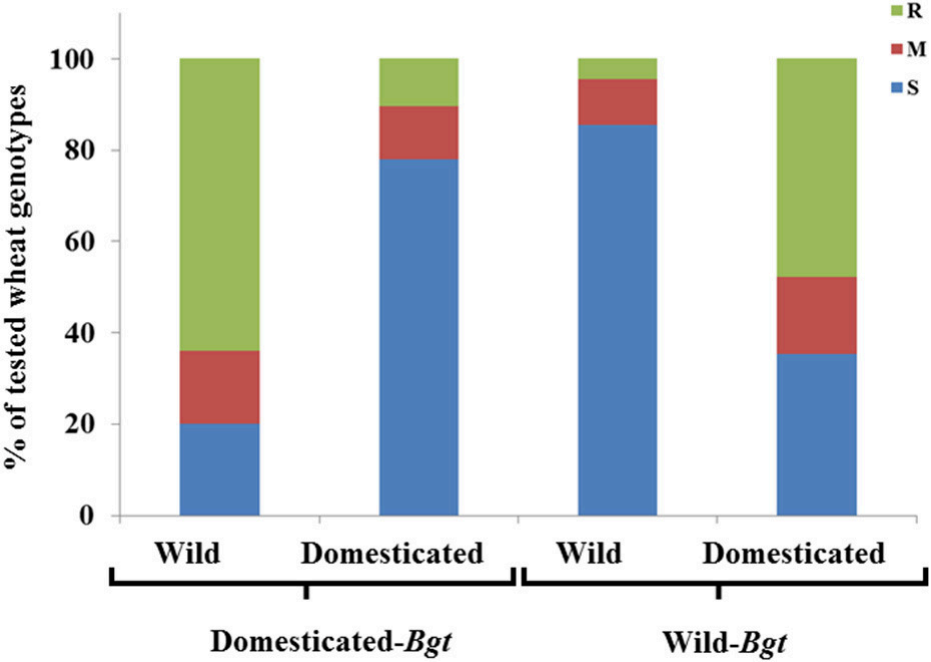
Distribution of qualitative disease resistance of wild and domesticated hosts. The distribution of phenotypic reactions of entries of wild and domesticated wheat lines [Resistant (R), Moderate (M), susceptible (s)], to *Bgt* isolates collected from domesticated wheat and wild emmer wheat.

Altogether, 1,692 interactions between wheat genotypes and *Bgt* isolates were qualitatively analyzed (Table 1, Figure 1, Figures S4, S5). When lines of specific wheat species were inoculated with *Bgt* isolates originating from the same species, low proportional rates of resistance to powdery mildew were detected, whereas higher resistance rates were evident when the pathogen isolates originated from different species. This trend was stronger when wild emmer wheat lines were exposed to isolates from domesticated hosts.

**Table 1:** The fraction of accessions (%), out of those tested (numbers), resistant to *Bgt* isolates collected from various wheat species.

***Bgt* isolate originated from**	**Bread wheat**	**Durum wheat**	**Wild emmer**
**HOST SPECIES**			
Bread wheat	6.1% (49)	4.1% (73)	68.0% (102)
Durum wheat	13.5% (74)	1.9% (103)	24.6% (146)
Wild emmer	48.0% (227)	53.9% (417)	4.1% (435)
Emmer wheat	27.7% (18)	21.7% (23)	20.0% (25)
χ^2^	63.9	211.5	325.9
*P*(χ^2^)	<0.0001	<0.0001	<0.0001

*Chi-square compare between categories within each column*.

Pooled data from all isolates and experiments can provide a general view of (wheat species × *Bgt* isolate) interactions (Table 2, Figure 1). A minute proportion (4.1%) of wild emmer accessions showed resistance when inoculated with *Bgt* isolates from wild emmer; the prominent χ^2^ value in this group [χ^2^ = 325.9; $\chi_{\left(\text{P}\right)}^{2}$ ≤ 0.0001] is due to the differing resistance proportions of the same lines when inoculated with *Bgt* isolates from bread wheat and durum wheat: 48 and 53.9%, respectively. Bread wheat genotypes were highly resistant (68%) to *Bgt* isolates from wild emmer, but very susceptible to *Bgt* isolates from cultivated wheats, with only 10.2% showing resistance [χ^2^ = 63.9; $\chi_{\left(\text{P}\right)}^{2}$ ≤ 0.0001]. Durum wheat varieties generally showed less resistance than bread wheats, but the general pattern was similar, with higher resistance against *Bgt* isolates from wild emmer. The frequency distribution of susceptibility among emmer wheat genotypes, the smallest group of wheat genotypes in the assay, does not show a differential pattern among the different origins of the *Bgt* isolates (Table 2).

**Table 2:** Analysis of variance of the quantitative resistance of a collection of wheat lines representing four species (*T. aestivum, T. durum, T. dicoccum*, and or *T. dicoccoides*) inoculated with *Bgt* isolates #15 and #66, #70 and #58, #97 and #63.

**ANALYSIS OF VARIANCE**^a^						
**Inoculation with**	***Bgt*****#15 and** ***Bgt*****#66**^b^		***Bgt*****#70 and** ***Bgt*****#58**^c^		***Bgt*****#97 and** ***Bgt*****#63**^d^	
**Source**	**d.f**.	**Mean square**	**d.f**.	**Mean square**	**d.f**.	**Mean square**
*Bgt* isolate (*Bgt*)	1	273.8^***^	1	5.0	1	115.0^**^
Species (S)	3	42.3	3	3.6	3	119.9
*Bgt* × S	3	361.6^***^	3	455.5^***^	3	654.6^***^
Line[Species]	56	36.4^***^	74	25.6^***^	82	46.3^***^
Model	63	67.8^***^	81	39.0^***^	89	69.2^***^
Experimental error	348	9.0	448	6.4	428	10.1
Total	411	−	529		517	−

a *The density of pustules was transformed to √(X+1) before ANOVA*.

b *Bgt isolate #15 was collected from durum wheat cultivar in Yavor Farm, North-Western Israel; Bgt isolate #66 was collected from wild emmer wheat in Ammiad natural reserve, North-Eastern Israel*.

c *Bgt isolate #70 was collected from common wheat cultivar in Kibutz Be'eri, South-Western Israel; Bgt isolate #58 was collected from wild emmer wheat in Ammiad natural reserve, North-Eastern Israel*.

d *Bgt isolate #97 was collected from durum wheat cultivar in Kibutz Negba, South-Western Israel; Bgt isolate #63 was collected from wild emmer wheat in Mt. Gilboa, North-Eastern Israel*.

**, ***, *and n.s. indicate significance at P < 0.001, 0.0001 or non-significant effect, respectively*.

### Quantitative phenotypic assay

**Figure 2:**
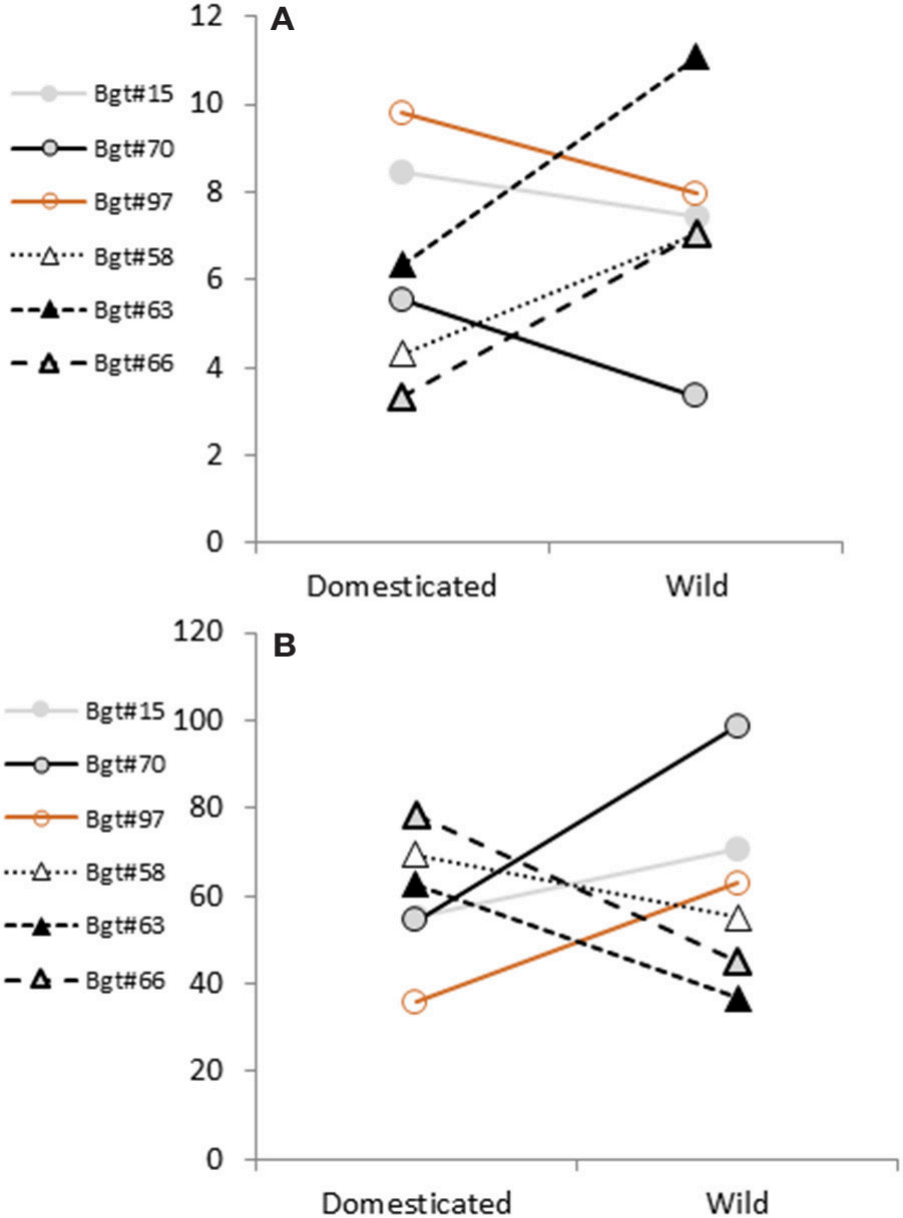
Mean comparison of quantitative disease responses of wild and domesticated hosts. Least square means **(A)** and CV values **(B)** showing the interaction between the pathogen (*Bgt* isolates) and host plants (domesticated/wild). Responses to *Bgt* from wild emmer host are shown as triangles and connected with dashed/dotted lines. Responses to *Bgt* from domesticated hosts bread wheat, durum wheat and wild emmer wheat are shown as open circles and connected with continuous lines.

Analysis of variance (ANOVA) of the three experiments is summarized in Table 2. R-square values for the three independent experiments ranged from 0.52 to 0.58, which highlights the proportion of the variance of disease symptoms across the differing wheat lines that was accounted for by the tested ANOVA model. The (wheat species × *Bgt* isolate) interaction was significant in all three experiments (Table 2, Figure S6). Wild emmer genotypes showed significantly higher disease severity when inoculated with isolates originating from wild emmer than with isolates collected from domesticated wheat (Figure 2, Figure S6). For example, in wild emmer accessions the mean severity value of *Bgt*#63, the most aggressive isolate, was manifested in appearance of 123 pustules/cm^2^ in wild emmer leaves, whereas in bread wheat genotypes the mean value was only 9.86 pustules/cm^2^ (original values were extracted by inversing the transformed data set).

A completely different pattern was evident when the domesticated lines were inoculated with *Bgt* originating from cultivated wheat fields: domesticated genotypes were heavily infected by inoculation with *Bgt* isolates collected from domesticated wheat species, as clearly exemplified by the high disease severity detected on bread wheat cultivars after inoculation with *Bgt*#70 isolate. In contrast, low levels of infection were evident in wild emmer wheat lines inoculated with powdery mildew isolates collected from domesticated wheat lines: disease severity values were 38.8, 10.1, and 39.1 pustules/cm^2^ for *Bgt*#15, *Bgt*#70, and *Bgt*#97, respectively (Figure S6).

In general, isolates sampled from modern wheat fields are showing a similar preference to domesticated host (Figure 2A). On the other hand, isolates sampled from wild wheat generated reciprocal reaction, showing reduced aggressiveness on domesticated germplasm and causing high disease severity in the wild material. To assess, variance estimates of disease severity CV values were compared (Figure 2B). Isolates from domesticated origin had a wider variance in pustules' density on wild emmer wheat. Their CV values where reduced when tested on domesticated hosts. All isolates originated from wild emmer wheat showed the exact reciprocal pattern.

**Figure 3:**
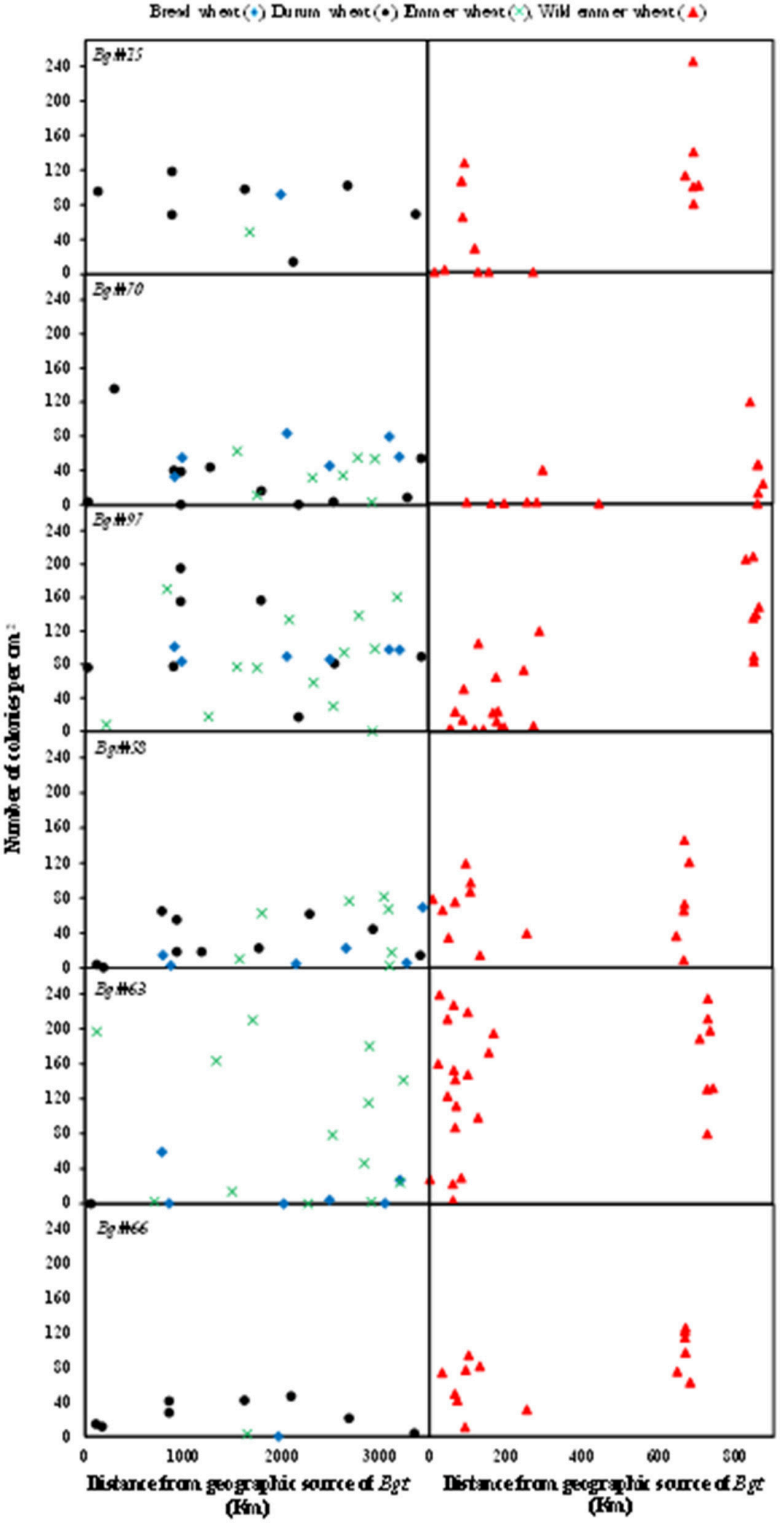
Association between geographic distance and disease response. Scatter chart showing the association between the geographic distances (km) of the host from the origin of the mildew isolate, and the number of mildew pustules/cm^2^ in the tests. Each row represents an individual mildew isolate that was used as inoculation source. The left column presents the disease severity of domesticated wheat lines. The right column represents disease reaction of wild emmer wheat accessions.

The geographical distances of the hosts from the sources of inoculum ranged between 0–3,500 and 0–900 km for domesticated and wild wheat genotypes, respectively (Figure 3). No association was observed between disease severity in the domesticated wheat genotypes and the distance from the geographical origin of the inoculum; disease severity values varied randomly along the geographical distance axis (Figure 3). Bread wheat genotypes showed low to very low disease severity, i.e., high resistance, when inoculated with isolates originating from wild wheat (Figure 3, left-hand side). In general, the wild emmer collection is geographically subdivided into Israeli (0–300 km from inoculum origin site) and eastern Turkish (600–900 km from inoculum origin site) groups (Figure S2). Both groups were divided by the Hierarchical clustering analysis into two distinct eco-geographic clusters. When inoculated with *Bgt* isolates from domesticated wheat the Israeli wild emmer accessions showed higher proportions of complete resistance (disease severity = 0) than the Turkish accessions (Figure 3, right-hand side). Disease severity in wild emmer after inoculation with *Bgt* isolates originating from wild hosts showed little or no differences between the Israeli and the Turkish subgroups, e.g., *Bgt*#58 (Figure 3, right-hand side).

## Discussion

The present study aims to characterize and investigate the wheat powdery mildew pathosystem and to test the hypothesis that disease responses of diverse host germplasm will correlate to the *Bgt* differentiation and adaptation to wild and domesticated host at the center of origin. Both qualitative and quantitative characterization have shown clear pattern of host differentiation in the response. The quantitative assay support biometrically the pattern of reciprocal hosts' responses to powdery mildew isolates originating from domesticated wheats.

### Qualitative phenotypic assay

A selected set of eight *Bgt* isolates was used for screening a diverse wheat collection by means of qualitative and quantitative phenotypic scoring. The qualitative assay which summarizes over 1,600 different (wheat genotype × *Bgt* isolate) interactions has shown strong and significant interaction between the origins of the isolates and the genetic sources of the tested wheat lines (Table 1, Figure 1, Figures S2, S3).

In general, *Bgt* isolates collected from cultivated wheat fields were more successful in attacking domesticated wheat genotypes than those of wild wheats. Wild emmer, in contrast, was significantly more resistant to these isolates. The isolates collected in the wild elicited reciprocal reactions, showing reduced virulence on domesticated germplasm and causing higher disease severity in the wild material (Figures 1, 2A). This finding is in full accordance to what was found in previous virulence reports of *Bgt* from Israel (Ben-David et al., 2016).

### Quantitative phenotypic assay

ANOVA of the quantitative test (Table 2) supported the finding of the qualitative assay by showing significant interaction between the pathogen (*Bgt* isolate) and the host (domesticated/wild wheat genotype). The symmetry of the reciprocal pattern was maintained despite the heterogeneity of the interaction slopes between isolates (Figure 2). These differences could be due to possible variance between the three independent experiments or to differing aggressiveness profiles of the different *Bgt* isolates. With regard to variance of disease severity a reciprocal trend was detected: all isolates originated from wild emmer showed wider variance in pustule density on domesticated than on wild hosts (in tests on wild emmer wheat the variance in pustule density was very small). On the other hand, the isolates originating from domesticated wheat showed an exactly reciprocal trend in CV values (Figure 2B). The factor responsible for high CV values is the high proportion of genotypes exhibiting complete resistance, i.e., IT = 0, which contributed to the overall within-group variance. Because this complete resistance is assumed to be conferred by major genes, it could serve as an additional evidence for host adaptation favoring accumulation of major resistance genes during the long host/pathogen co-evolution process. Together with the disease severity data, this finding strongly supports the differentiation between *Bgt* isolates from wild and domesticated origins, respectively, which was implied by Eshed et al. (1994). Likewise, Krupinsky (1997) showed that *Stagonospora* isolates from wild origins had lower aggressiveness than those from domesticated ones. The results obtained in the present study are also in full accord with the findings of Frenkel et al. (2008) in the *Didymella rabiei/*chickpea pathosystem. *Didymella rabiei* isolates from domesticated origin were significantly more aggressive on domesticated chickpea, than isolates from wild origin. In contrast but similarly to what we have found in our study on the wild host, *C. judaicum*, isolates from wild origin were generally more aggressive than isolates from domesticated origin. However, host specificity is not always detected. A recent investigation of a similar legume pathosystem of *Peyronellaea pinodes* of *Pisum* sp. suggested that Israel might be inhabited with a single metapopulational of the pathogen with no clear differentiation of wild and cultivated host (Golani et al., 2016).

The wild emmer *Bgt* isolates attacked the hexaploid wheat hardly at all, and attacked the domesticated tetraploid wheat only to an intermediate extent. The durum wheat *Bgt* attacked the full range of wheat species tested, but the wild tetraploid wheat to a somewhat lesser extent. The bread wheat *Bgt* was highly virulent on the hexaploid wheat, moderately virulent on domesticated tetraploid wheat, and was much less virulent on wild emmer. This quantitative reciprocal pattern provides solid support for the interpretations of Eshed and Wahl (1970) and Wyand and Brown (2003) regarding *formae speciales* in *Bgt* and in a more directed manner for the recently published evidence for *bgt* specialization (Ben-David et al., 2016; Menardo et al., 2016).

We have also examined the association between geographic distance and virulence. The *Bgt* capability to produce huge numbers of spores that are wind-borne from one susceptible host to another, is countered by the fact that long-distance dissemination of *Bgt* is limited to hundreds of kilometers on a multiseasonal time-scale and might involve a pronounced role of human-dependent dispersal of cleistothecia, (e.g., *Bgt* USA population; Brown and Hovmøller, 2002; Parks et al., 2009). The geographical distribution of the cultivated wheat varieties tested is much wider than that of the wild emmer samples tested. In addition, the wild emmer samples are derived from two distinct areas, which leads to a binomial distribution rather than a geographically continuous distribution of distances (Figure 3). Nevertheless, the results show that bread wheat cultivars exhibit high or complete resistance to *Bgt* derived from wild wheat populations. The higher portion of complete resistance of the Israeli wild emmer compared with the Turkish subgroup might imply that local adaptation of the host took place in the Israeli natural habitats. The high proportion of the wild accessions that exhibited complete resistance (disease severity = 0) when inoculated with *Bgt* from cultivated wheat might imply the involvement of R-genes in this local adaptation process. This trend was less evident when inoculation was done with *Bgt* from wild wheat populations which might imply wild host specialization (Figure 3). A recently developed demographic–genetic simulation model assumes co-existence of crop specialists and wild host specialists within a pathogen population. Based on the model this co-existence can occur under various scenarios of pathogen demography and pathogen dispersal (Papaïx et al., 2015). Our recent finding regarding *Bgt* differentiation based on host origin (see also Ben-David et al., 2016) may provide strong empirical support to such agro-ecological landscape models.

## Conclusions

The reciprocal pattern, which was expressed throughout the interactions of wild compared with domesticated host species tested with eight *Bgt* isolates from various origins, probably results from long-term plant/pathogen co-evolution. Indeed, recent molecular analyses of widely diverse *Bgt* isolates obtained from sympatric wild and domesticated hosts have shed light upon ongoing differentiation (Ben-David et al., 2016; Menardo et al., 2016). Consistently with previous findings (Eshed et al., 1994) the present results indicate that in its center of origin, the wheat *forma specialis* of *B. graminis* might be in the process of diverging into two populations of mildew, one of emmer and one of domesticated wheat.

## Author contributions

RB-D, AD, ZP, and TF: Designed the experiments; RB-D, AD and ZP: Conducted the experiment; RB-D, AD, and ZP: Analyzed data and wrote the paper; All authors read and approved the manuscript.

### Conflict of interest statement

The authors declare that the research was conducted in the absence of any commercial or financial relationships that could be construed as a potential conflict of interest.

## Supplementary material

Figure S1 Schematic chart illustrating the evolutionary relations between wheat species included in this study.

Figure S2 A graphical representation of the experimental plan.

Figure S3 Geographic distribution of wheat collection. Geographic distribution map of wheat lines collection sites across the Mediterranean and west Asia. Sites from which accessions originated are marked by black points. The pink circle represents Israel, the most intensively sampled region (Map generated by Map Send).

Figure S4 Distribution of qualitative disease reaction to *Bgt* from domesticated wheat. The distribution of phenotypic reactions of entries of wild and domesticated wheat lines [Resistant (R), Moderate (M), Susceptible (S)], to *Bgt* isolates collected from domesticated wheat. ^*^, ^**^, or ^***^ indicate χ^2^ significance level *P* ≤ 0.05, 0.01, or 0.001, respectively.

Figure S5 Distribution of qualitative disease reaction to *Bgt* from wild wheat. The distribution of phenotypic reactions of entries of wild and domesticated wheat lines [Resistant (R), Moderate (M), Susceptible (S)] to *Bgt* isolates collected from wild wheat. ^*^, ^**^, and ^***^ indicate χ^2^ significance level of *P* ≤ 0.05, 0.01, or 0.001, respectively.

Figure S6 Comparison of disease responses of wild and domesticated host's species. Comparison of means of disease severity (Transformed no. of mildew pustules/cm^2^) between wheat species inoculated with single *Bgt* isolates. For all isolates Tukey's LSD test was applied to the results. No result for *Bgt*#66 on bread wheat is presented because there were no symptoms of powdery mildew. **(A)** Inoculation with *Bgt*#15 and *Bgt*#66; **(B)** Inoculation with *Bgt*#70 and *Bgt*#58; **(C)** Inoculation with *Bgt*#97 and *Bgt*#63.

Table S1 Wheat accession name, *Triticum* species, country of origin and site of collection.

Table S2 The reactions of *Pm* differential wheat lines to a set of seven *Bgt* isolates originating from various wheat species.

Table S3 Phenotypic characterization of the two parental lines Langdon and G18-16 for powdery mildew resistance to 47 *Bgt* isolates.

Supplementary File Statistical analyses.
