# Supplementary material for: Reciprocal Hosts' Responses to Powdery Mildew Isolates Originating from Domesticated Wheats and Their Wild Progenitor
Source: Front Plant Sci. 2018 Feb 23;9:75. doi: 10.3389/fpls.2018.00075 (PMC5829517; doi:10.3389/fpls.2018.00075)
Supplement: Figure S1 — Schematic chart illustrating the evolutionary relations between wheat species included in this study. [file Image1.PDF]

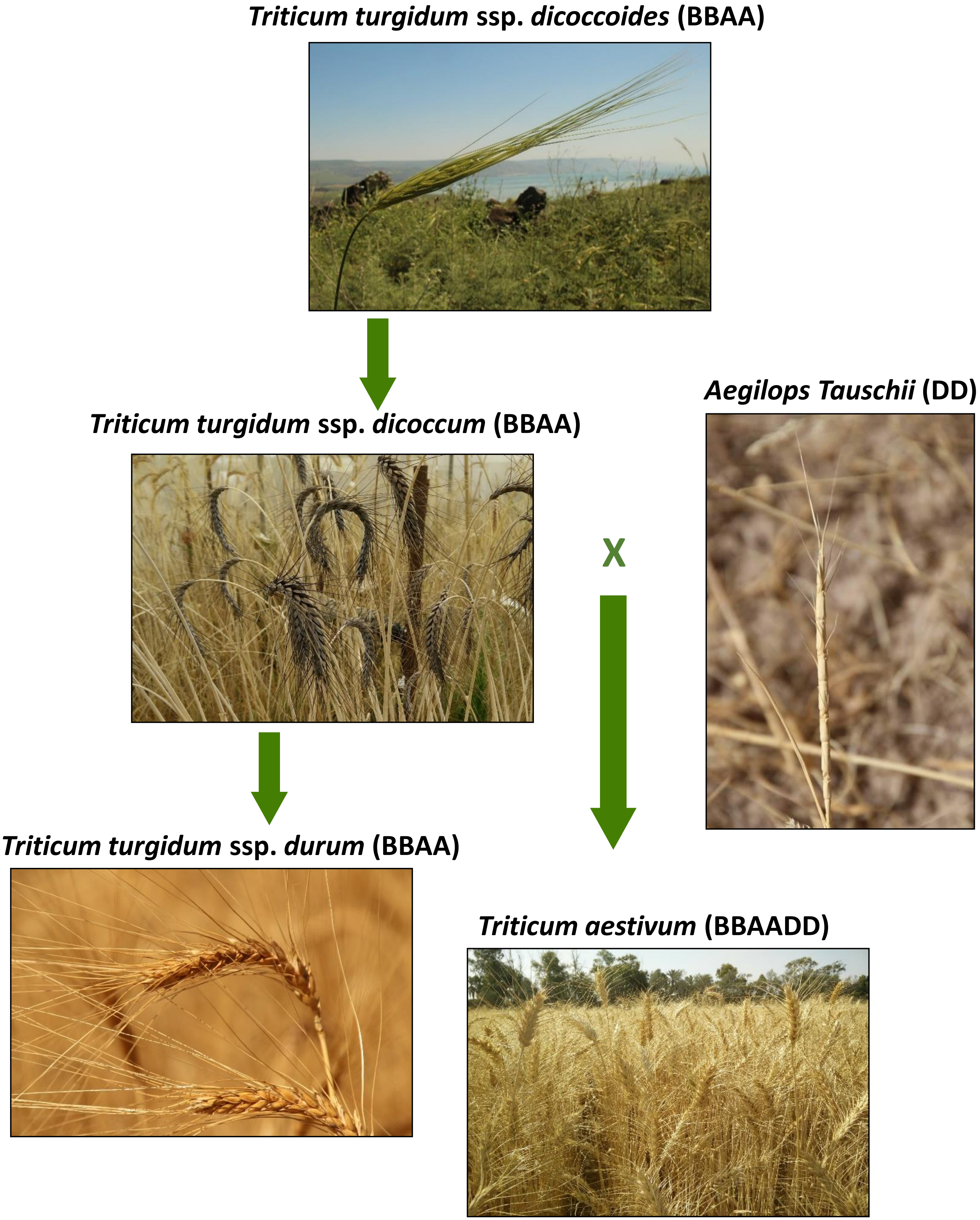
Triticum turgidum ssp. *dicoccoides* (BBAA)
Triticum turgidum ssp. *dicoccum* (BBAA)
Aegilops Tauschii (DD)
X
Triticum turgidum ssp. *durum* (BBAA)
Triticum aestivum (BBAADD)
