# Supplementary figures and images for: Reciprocal Hosts' Responses to Powdery Mildew Isolates Originating from Domesticated Wheats and Their Wild Progenitor
Source: Front Plant Sci. 2018 Feb 23;9:75. doi: 10.3389/fpls.2018.00075 (PMC5829517; doi:10.3389/fpls.2018.00075)

# Phenotypic test of diverse collection of wheat germplasm

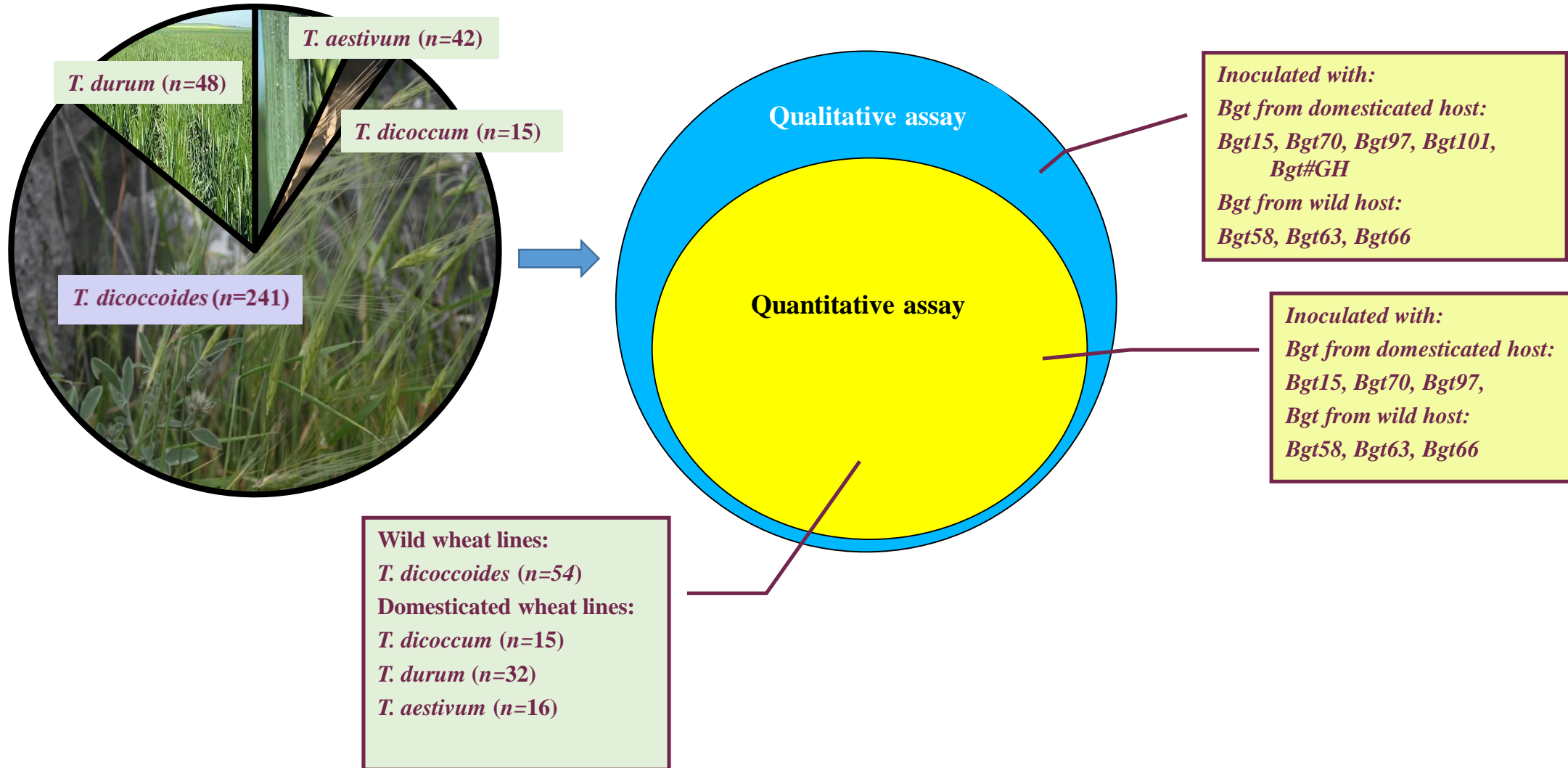

Supplement: Figure S2 — A graphical representation of the experimental plan. [file Image2.PDF]

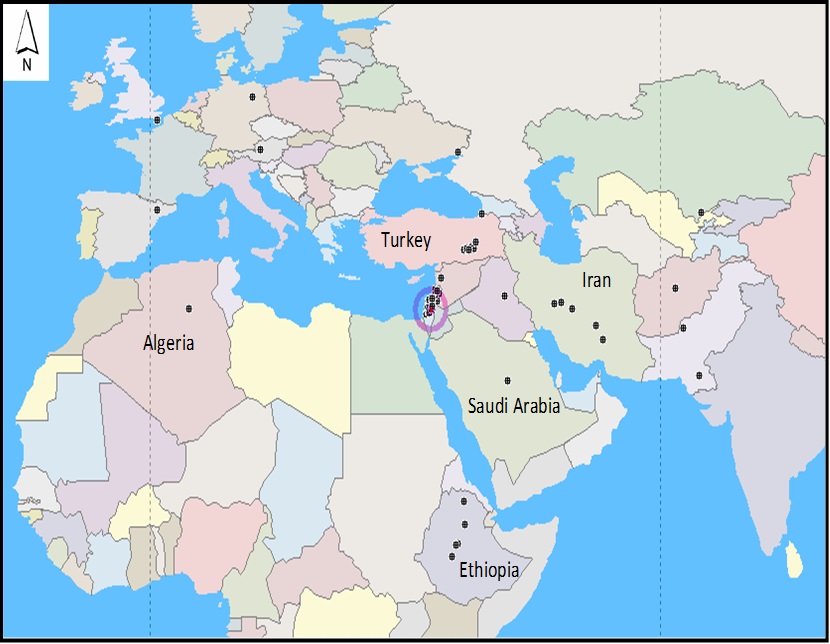

Supplement: Figure S3 — Geographic distribution of wheat collection. Geographic distribution map of wheat lines collection sites across the Mediterranean and west Asia. Sites from which accessions originated are marked by black points. The pink circle represents Israel, the most intensively sampled region (Map generated by Map Send). [file Image3.JPEG]

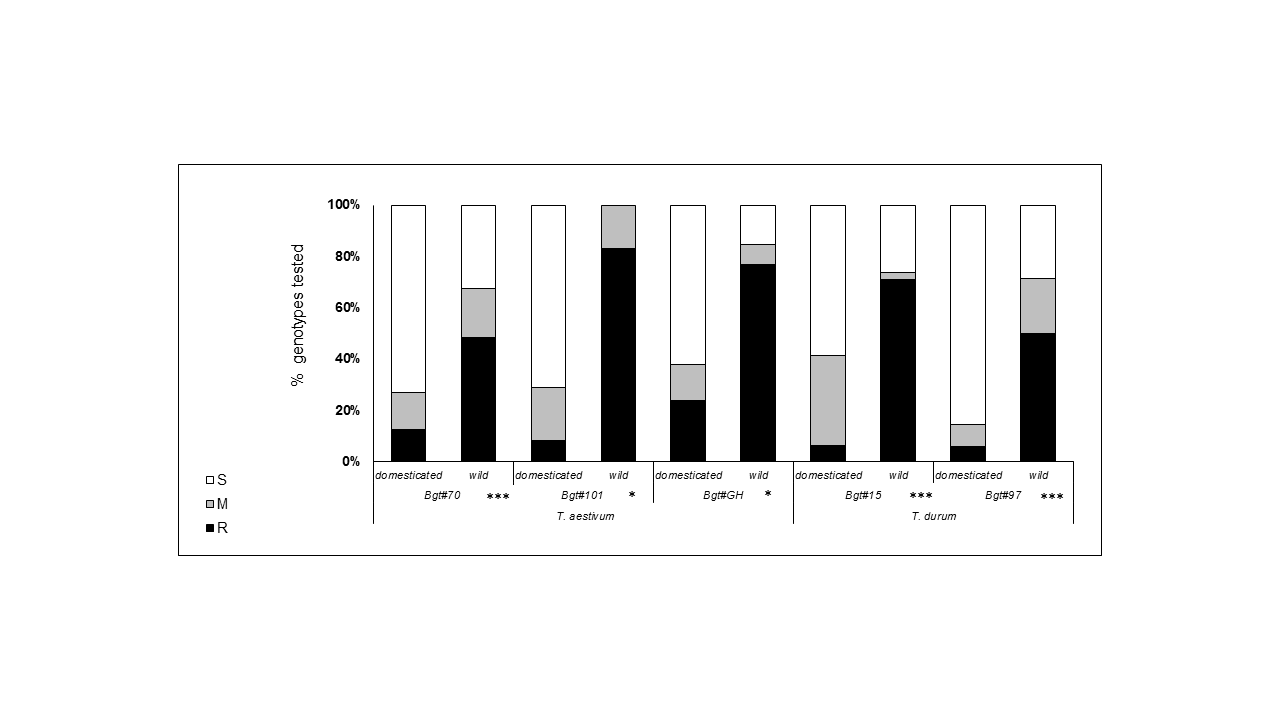

Supplement: Figure S4 — Distribution of qualitative disease reaction to Bgt from domesticated wheat. The distribution of phenotypic reactions of entries of wild and domesticated wheat lines [Resistant (R), Moderate (M), Susceptible (S)], to Bgt isolates collected from domesticated wheat. *, **, or *** indicate χ2 significance level P ≤ 0.05, 0.01, or 0.001, respectively. [file Image4.tif]

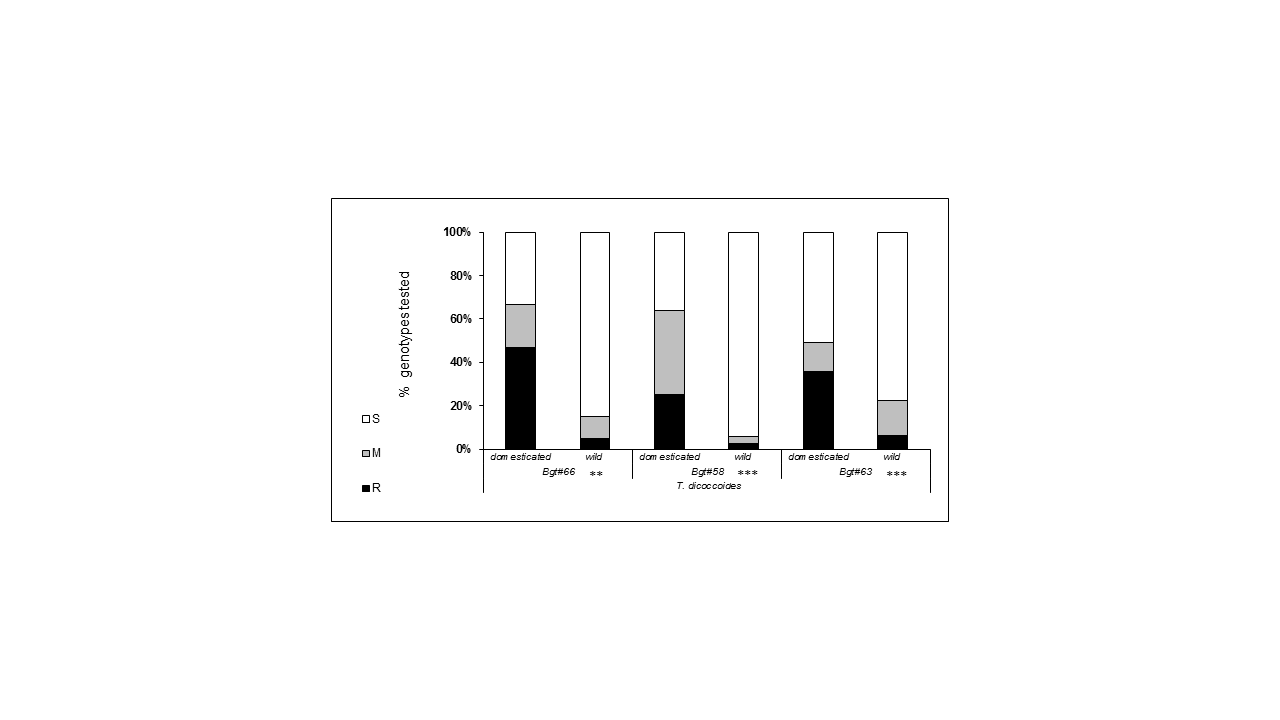

Supplement: Figure S5 — Distribution of qualitative disease reaction to Bgt from wild wheat. The distribution of phenotypic reactions of entries of wild and domesticated wheat lines [Resistant (R), Moderate (M), Susceptible (S)] to Bgt isolates collected from wild wheat. *, **, and *** indicate χ2 significance level of P ≤ 0.05, 0.01, or 0.001, respectively. [file Image5.tif]

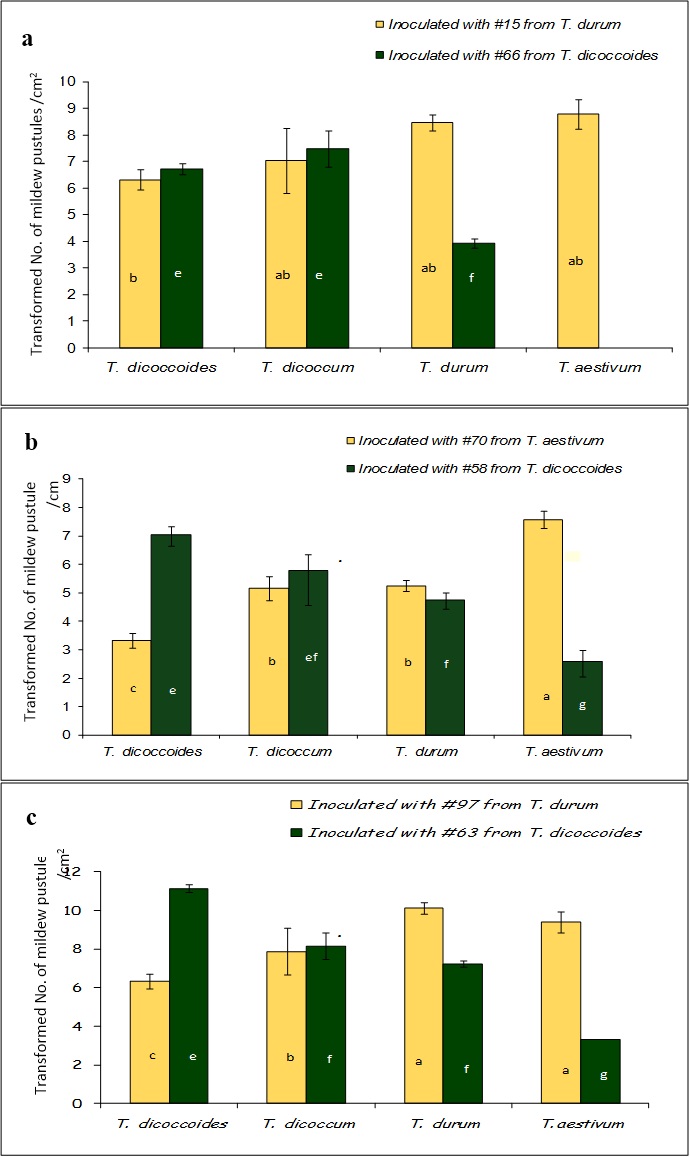

Supplement: Figure S6 — Comparison of disease responses of wild and domesticated host's species. Comparison of means of disease severity (Transformed no. of mildew pustules/cm2) between wheat species inoculated with single Bgt isolates. For all isolates Tukey's LSD test was applied to the results. No result for Bgt#66 on bread wheat is presented because there were no symptoms of powdery mildew. (A) Inoculation with Bgt#15 and Bgt#66; (B) Inoculation with Bgt#70 and Bgt#58; (C) Inoculation with Bgt#97 and Bgt#63. [file Image6.JPEG]
