# Supplementary material for: Reciprocal Hosts' Responses to Powdery Mildew Isolates Originating from Domesticated Wheats and Their Wild Progenitor
Source: Front Plant Sci. 2018 Feb 23;9:75. doi: 10.3389/fpls.2018.00075 (PMC5829517; doi:10.3389/fpls.2018.00075)
Supplement: Table S1 — Wheat accession name, Triticum species, country of origin and site of collection. [file Table1.DOC]

| **Table S1. Wheat accession name, *Triticum* specie, country of origin and site of collection** | | | | | | |
| --- | --- | --- | --- | --- | --- | --- |
| **Accession Name** | **Species** | **Origin** | | **Site** | **LON_EX** | **LAT_EX** |
| 20-49 | *T. turgidum ssp. dicoccoides* | Israel | | Taiyba | 35.30 | 31.95 |
| 23-24 | *T. turgidum ssp. dicoccoides* | Israel | | J'aba | 35.07 | 31.67 |
| 24-39 | *T. turgidum ssp. dicoccoides* | Israel | | Amirim | 35.45 | 32.92 |
| 26-33 | *T. turgidum ssp. dicoccoides* | Israel | | Achihood | 35.17 | 32.90 |
| 28-53 | *T. turgidum ssp. dicoccoides* | Israel | | Beit Oren | 35.00 | 32.72 |
| 32-35 | *T. turgidum ssp. dicoccoides* | Israel | | Yabad | 35.15 | 32.43 |
| 33-8 | *T. turgidum ssp. dicoccoides* | Israel | | Givat Koach | 34.92 | 32.02 |
| BR 1/1 | *T. turgidum ssp. dicoccoides* | Israel | | Camp Brosh | 35.52 | 32.30 |
| BR 1/2 | *T. turgidum ssp. dicoccoides* | Israel | | Camp Brosh | 35.52 | 32.30 |
| dic115 | *T. turgidum ssp. dicoccoides* | Israel | | Safad on road to Rosh Pinna | 35.48 | 32.97 |
| dic42 | *T. turgidum ssp. dicoccoides* | Israel | |  | 35.75 | 31.50 |
| dic48 | *T. turgidum ssp. dicoccoides* | Israel | | Bat Shelomo | 35.00 | 32.58 |
| dic79 | *T. turgidum ssp. dicoccoides* | Israel | | Bet Meir | 35.03 | 31.80 |
| I69 | *T. turgidum spp. dicoccum* | Syrian Arab Republic | | Der'a Provinz | 36.12 | 32.80 |
| KH 4/1 | *T. turgidum ssp. dicoccoides* | Israel | | Kokhav Hayarden | 35.53 | 32.60 |
| ME 2/2 | *T. turgidum ssp. dicoccoides* | Israel | | Mhale Efraim | 35.38 | 32.07 |
| P-9 | *T. turgidum ssp.durum* | Israel | | Abu Fashit | 34.75 | 31.50 |
| TZ74 | *T. turgidum ssp. dicoccoides* | Israel | | kohav hasahar | 35.33 | 31.95 |
| C111 | *T. aestivum* | Iran | | 1940, Kerman, Bahamabad (=Rafsanjan) | 56.02 | 30.37 |
| C88 | *T. turgidum ssp. durum* | Iran | | 1940, Gandumi Andojird. Kerman, Chaharfarsakh | 56.97 | 29.05 |
| **Table S1. (Continued)** | | | | | | |
| **Accession Name** | **Species** | **Origin** | **Site** | | **LON_EX** | **LAT_EX** |
| dico123 | *T. turgidum ssp. dicoccoides* | Syria | Syria, Damascus Province, may Saloum | | 36.32 | 33.52 |
| I80 | *T. turgidum spp. dicoccum* | Iran | Shahr-Kord | | 50.85 | 32.55 |
| MM3/2 | *T. turgidum ssp. dicoccoides* | Israel | Mhale Merav | | 36.78 | 32.43 |
| N106 | *T. turgidum ssp. durum* | Iraq | Bagdad | | 44.60 | 33.30 |
| N107 | *T. turgidum ssp. durum* | Pakistan | Sind | | 69.00 | 25.50 |
| N108 | *T. turgidum ssp. durum* | Iraq | Bagdad | | 44.60 | 33.30 |
| N2 | *T. aestivum* | Pakistan | Baluchistan, 5100 | | 67.00 | 30.20 |
| N53 | *T. turgidum spp. dicoccum* | Morocco | 250 | | 5.00 | 32.00 |
| N55 | *T. turgidum spp. dicoccum* | Saudi Arabia |  | | 45.00 | 25.00 |
| N66 | *T. turgidum spp. dicoccum* | Iran | landrace, 1955 | | 53.00 | 32.00 |
| N99 | *T. turgidum spp. dicoccum* | Iran | esfahan | | 51.68 | 32.68 |
| C103 | *T. turgidum ssp. durum* | Ethiopia | 1955 Ethiopia , Shewa, Addis Abeba | | 38.75 | 9.02 |
| N61 | *T. turgidum spp. dicoccum* | Ethiopia | welo | | 39.63 | 11.13 |
| N64 | *T. turgidum spp. dicoccum* | Ethiopia | landrace, 1951 | | 38.00 | 8.00 |
| N92 | *T. turgidum spp. dicoccum* | Ethiopia | Tigre | | 39.53 | 13.38 |
| N93 | *T. turgidum spp. dicoccum* | Ethiopia | shewa | | 38.48 | 9.07 |
| C101 | *T. turgidum ssp. durum* | Lebanon | 1948, Lebanon, Bekaa. Chtura | | 35.85 | 33.82 |
| dic112 | *T. turgidum ssp. dicoccoides* | Syria | Damascus Province | | 36.08 | 33.73 |
| dic114 | *T. turgidum ssp. dicoccoides* | Israel | south of Rosh Pinna | | 35.53 | 32.97 |
| **Table S1 (Continued)** | |  |  | |  |  |
| **Accession Name** | **Species** | **Origin** | **Site** | | **LON_EX** | **LAT_EX** |
| dic53 | *T. turgidum ssp. dicoccoides* | Israel | safad | | 35.50 | 32.97 |
| dic97 | *T. turgidum ssp. dicoccoides* | Israel | Rosh Pinna | | 35.53 | 32.97 |
| dico70 | *T. turgidum ssp. dicoccoides* | Lebanon | Rashaya | | 35.83 | 33.50 |
| dico145 | *T. turgidum ssp. dicoccoides* | Syria | Syria; Hama' near Birin | | 36.65 | 34.98 |
| I21 | *T. turgidum ssp. dicoccoides* | Turkey | Diyarbakir | | 40.10 | 37.75 |
| I71 | *T. turgidum spp. dicoccum* | Turkey | urfa(same as N30) | | 39.50 | 37.72 |
| N100 | *T. turgidum ssp. durum* | Uzbekistan | tashkent | | 69.30 | 41.33 |
| N112 | *T. turgidum ssp. durum* | Turkey | Diyarbakir | | 40.33 | 37.97 |
| N22 | *T. turgidum ssp. dicoccoides* | Turkey | 44 km west of Diyarbakir in the Karacadag | | 39.82 | 37.83 |
| N23 | *T. turgidum ssp. dicoccoides* | Turkey | 51 km west of Diyarbakir in the Karacadag | | 39.77 | 37.80 |
| N27 | *T. turgidum ssp. dicoccoides* | Turkey | 51 km west of Diyarbakir in the Karacadag | | 39.77 | 37.80 |
| N28 | *T. turgidum ssp. dicoccoides* | Turkey | 33.6 km west of Diyarbakir in the Karacadag | | 39.88 | 37.87 |
| N3 | *T. aestivum* | Turkey | Diyarbakir, kirmizi | | 40.67 | 37.85 |
| N30 | *T. turgidum ssp. dicoccoides* | Turkey | 20.2 km east of Siverek | | 39.50 | 37.72 |
| N32 | *T. turgidum ssp. dicoccoides* | Turkey | 36.2 km west of Diyarbakir in the Karacadag | | 39.87 | 37.88 |
| N35 | *T. turgidum ssp. dicoccoides* | Turkey | 52.6 km west of Diyarbakir in the Karacadag | | 39.77 | 37.78 |
| N37 | *T. turgidum ssp. dicoccoides* | Turkey | 52.5 km west of Diyarbakir in the Karacadag | | 39.77 | 37.78 |
| N4 | *T. aestivum* | Turkey | Diyarbakir, 5721 | | 41.02 | 38.50 |
|  |  |  |  | |  |  |
| **Table S1 (Continued)** | |  |  | |  |  |
| Accession Name | Species | Origin | Site | | **LON_EX** | **LAT_EX** |
| dico184 | *T. turgidum ssp. durum* | Ukraine | Landrace | | 38.78 | 47.22 |
| I35 | *T. turgidum spp. dicoccum* | Italy | Villa Bigioni | | 12.98 | 52.58 |
| N114 | *T. turgidum ssp. Turgidum* | Turkey | Artvin | | 41.77 | 41.27 |
| N14 | *T. aestivum spp. spelta* | Austria | Roter Begranter Tiroler | | 14.00 | 47.50 |
| N15 | *T. aestivum spp. spelta* | Belgium | Namur | | 5.12 | 50.27 |
| N56 | *T. turgidum spp. dicoccum* | Afghanistan |  | | 66.00 | 34.00 |
| N60 | *T. turgidum spp. dicoccum* | Spain | Zaragoza | | 1.00 | 41.58 |
| dic100 | *T. turgidum ssp. dicoccoides* | Israel | ramla jank. | | 35.45 | 31.83 |
