# Supplementary material for: Reciprocal Hosts' Responses to Powdery Mildew Isolates Originating from Domesticated Wheats and Their Wild Progenitor
Source: Front Plant Sci. 2018 Feb 23;9:75. doi: 10.3389/fpls.2018.00075 (PMC5829517; doi:10.3389/fpls.2018.00075)
Supplement: Table S2 — The reactions of Pm differential wheat lines to a set of seven Bgt isolates originating from various wheat species. [file Table2.DOC]

| **Table S2** The reactions of *Pm* differential wheat lines to a set of seven *Bgt* isolates originating from various wheat species. | | | | | | | | | | | | | | | | | | | | | | | | | | |
| --- | --- | --- | --- | --- | --- | --- | --- | --- | --- | --- | --- | --- | --- | --- | --- | --- | --- | --- | --- | --- | --- | --- | --- | --- | --- | --- |
| **Genotype** | **Genetic source** | **Cultivar/genotype** | **Accession*#^a^*** | ***Bgt* Isolate** | | **#58** | | | **#63** | | | **#66** | | **#15** | | | **#70** | | | **#97** | | | | **#101** | | |
|  |  |  |  | Host species | | Wild emmer | | | Wild emmer | | | Wild emmer | | Durum wheat | | | Bread wheat | | | Durum wheat | | | | Bread wheat | | |
|  |  |  |  | Location | | Amiad | | | Gilboa | | | Amiad | | Yavor | | | Be'eri | | | Negba | | | Nahal Oz | | | |
|  |  |  |  | Cultivar | | - | | | - | | | - | | Inbar | | | Deganit | | | M | | | Dari'el | | | |
| ***Pm1a*** | *T. aestivum* | Axminister/8*Cc | CItr14114 | | S | | S | | | R | | | S | | S | | | | S | | S | | | | |  |
| ***Pm1b*** | *T. monnococum* | MocZlatka | -- | | R | | R | | | R | | | R | | S | | | | S | | S | | | | |  |
| ***Pm2*** | *Aegilops squarrosa* | Ulka/8*Cc | CItr14118 | | R | | R | | | R | | | S | | S | | | | S | | S | | | | |  |
| ***Pm3a*** | *T. aestivum* | Asosan'/'8*Ce | CItr14120 | | R | | R | | | R | | | S | | S | | | | S | | S | | | | |  |
| ***Pm3b*** | *T. aestivum* | Chul/'8*Ce | CItr14121 | | R | | R | | | R | | | R | | S | | | | S | | S | | | | |  |
| ***Pm3c*** | *T. aestivum* | Sonora/8*Cc | CItr14122 | | S | | R | | | R | | | S | | S | | | | S | | S | | | | |  |
| ***Pm3d*** | *T. aestivum* | Kolibri | -- | | S | | R | | | R | | | R | | S | | | | S | | R | | | | |  |
| ***Pm4a*** | *T. dicoccum* | Khapli/8*Cc | CItr14123 | | R | | S | | | R | | | R | | R | | | | R | | S | | | | |  |
| ***Pm4b*** | *T. carthlicum* | Weihenstephan | -- | | R | | R | | | R | | | S | | R | | | | S | | R | | | | |  |
| ***Pm5a*** | *T. dicoccum* | Hope /8* Chancellor | CItr14125 | | S | | S | | | R | | | S | | S | | | | S | | S | | | | |  |
| ***Pm5b*** | *T. aestivum* | Ibis | -- | | S | | S | | | R | | | S | | S | | | | S | | S | | | | |  |
| ***Pm6*** | *T. timopheevii* | TP114/2*Starke | CItr15888 | | S | | S | | | R | | | S | | S | | | | S | | S | | | | |  |
| ***Pm7*** | *S. cereale* | Transec | -- | | S | | S | | | R | | | S | | S | | | | S | | S | | | |  | |
| ***Pm8*** | *S. cereale* | Disponent | -- | | R | | R | | | R | | | S | | | R | | | S | | S | | | |  | |
| ***Pm17*** | *S. cereale* | Amigo | -- | | R | | R | | | R | | | R | | | S | | | S | | S | | | |  | |
| ***Pm22*** | *T. aestivum* | Virest | -- | | S | | | S | | | - | | S | | | S | | S | | | | S | | |  | |
| ***Pm2+Pm6*** | -- | Mads Huntsman | -- | | R | | | R | | | R | | R | | | R | | S | | | | R | | |  | |
| ***Pm1+Pm2+Pm9*** | -- | Normandie | -- | | R | | | R | | | R | | R | | | S | | S | | | | S | | |  | |
| *^a^* National Plant Germplasm System (http://www.ars-grin.gov/npgs/acc/acc_queries.html). | | | | | | | | | | | | | | | | | | | | | | | | | | |
