# Supplementary material for: Reciprocal Hosts' Responses to Powdery Mildew Isolates Originating from Domesticated Wheats and Their Wild Progenitor
Source: Front Plant Sci. 2018 Feb 23;9:75. doi: 10.3389/fpls.2018.00075 (PMC5829517; doi:10.3389/fpls.2018.00075)
Supplement: Table S3 — Phenotypic characterization of the two parental lines Langdon and G18-16 for powdery mildew resistance to 47 Bgt isolates. [file Table3.doc]

**Table S3.** Phenotypic characterization of the two parental lines Langdon and G18-16 for powdery mildew resistance to 47 *Bgt* isolates

| **Isolate #** | **Collection site** | | | | **Wheat species collected from** | | **Wheat**  **cultivar** | | | **Infection type^a^** | | | | | |
| --- | --- | --- | --- | --- | --- | --- | --- | --- | --- | --- | --- | --- | --- | --- | --- |
|  | **country** | **site** | | |  |  |  |  |  | **G18-16** | | | **Langdon** | | |
| **1** | Israel | Hula Valley | | | *T. aestivum* | | Dariel | | | R^a^ | | | S | | |
| **4** | Israel | Hula Valley | | | *T. aestivum* | | Dganit | | | S | | | S | | |
| **6** | Israel | Hula Valley | | | *T. aestivum* | | Atir | | | R | | | S | | |
| **7** | Israel | Hula Valley | | | *T. aestivum* | | Shapir | | | S | | | S | | |
| **8** | Israel | Hula Valley | | | *T. durum* | | Bareket | | | R | | | S | | |
| **9** | Israel | Hula Valley | | | *T. aestivum* | | 652 | | | S | | | S | | |
| **13** | Israel | Hula Valley | | | *T. aestivum* | | 519 | | | R | | | S | | |
| **15** | Israel | Yavor | | | *T. durum* | | Inbar | | | R | | | S | | |
| **16** | Israel | Nahal Oz | | | *T. durum* | | Inbar | | | R | | | S | | |
| **20** | Israel | Ein Hanatziv | | | *T. durum* | | Inbar | | | R | | | S | | |
| **25** | Israel | Bet Dagan | | | *T. durum* | | Inbar | | | R | | | R | | |
| **29** | Israel | Ein Hanatziv | | | *T. aestivum* | | Shapir | | | R | | | S | | |
| **30** | Israel | Talmei Yafe | | | *T. aestivum* | | Shapir | | | R | | | S | | |
| **33** | Israel | Erez | | | *T. aestivum* | | Dganit | | | S | | | S | | |
| **36** | Israel | Lahav | | | *T. aestivum* | | Dganit | | | R | | | S | | |
| **37** | Israel | Nahal Oz | | | *T. aestivum* | | Dganit | | | R | | | S | | |
| **43** | Israel | Yesodot | | | *T. aestivum* | | Bet Hashita | | | R | | | S | | |
| **44** | Israel | Negev | | | *T. aestivum* | | Miriam | | | R | | | S | | |
| **47** | Israel | Sa'ad | | | *T. aestivum* | | Bet Lehem | | | R | | | R | | |
| **50** | Israel | Nahal Oz | | | *T. aestivum* | | Barkai | | | R | | | S | | |
| **52** | Israel | Dir El Balakh | | | - | | - | | | R | | | S | | |
| **58** | Israel | Ammiad | | | *T. dicoccoides* | | 58 | | | S | | | S | | |
| **61** | Israel | Tabigha | | | *T. durum^b^* | | ZB | | | S | | | S | | |
| **63** | Israel | Gilbboa | | | *T. dicoccoides* | | - | | | S | | | S | | |
| **64** | Israel | Karei Deshe | | | *T. dicoccoides* | | - | | | S | | | S | | |
| **66** | Israel | Ammiad | | | *T. dicoccoides* | | - | | | S | | | R | | |
| **67** | Israel | Lahav | | | *T. durum* | | Inbar | | | R | | | S | | |
| **68** | Israel | Bet Dagan | | | *T. aestivum* | | Bet- Hashita | | | S | | | S | | |
| **70** | Israel | Beeri | | | *T. aestivum* | | Dganit | | | R | | | S | | |
| **91** | Israel | Sde Eliahu | | | *T. durum* | | Inbar | | | R | | | S | | |
| **92** | Israel | Tel Aviv | | | *T. dicoccoides* | | - | | | R | | | S | | |
| **94** | Israel | Ein Hanatziv | | | *T. aestivum* | | Dganit | | | R | | | S | | |
| **95** | Israel | Ein Hanatziv | | | *T. durum* | | Bareket | | | S | | | S | | |
| **96** | Israel | Negba | | | *T. aestivum* | | M50 | | | R | | | S | | |
| **97** | Israel | Negba | | | *T. durum* | | M | | | S | | | S | | |
| **101** | Israel | Nahal Oz | | | *T. aestivum* | | Dariel | | | R | | | S | | |
| **103** | Israel | Ammiad | | | *T. dicoccoides* | | - | | | R | | | R | | |
| **106** | Israel | Nahal Oz | | | *T. aestivum* | | Atir | | | R | | | S | | |
| **107** | Israel | Nahal Oz | | | *T. aestivum* | | Shapir | | | R | | | R | | |
| **108** | Israel | Nahal Oz | | | *T. aestivum* | | Bareket | | | R | | | S | | |
| **109** | Israel | Tel Aviv | | | *T. dicoccoides* | | - | | | S | | | S | | |
| **113** | Israel | Ammiad? | | | *T. dicoccoides* | | - | | | R | | | S | | |
| **96229** | Switzerland | Ellighausen-  Kloten Rd. | | | *T. aestivum*^b^ | - | | R | | | | S | | |  |
| **96224** | Switzerland | Winterthur-  Kloten Rd. | | | *T. aestivum*^b^ | - | | R | | | | S | | |  |
| **96236** | Switzerland | Oensingen-  Schoetz Rd. | | *T. aestivum*^b^ | | | - | | R | | S | | |  | |
| **96244** | Switzerland | Coppet -  Yverdon Rd. | *T. aestivum*^b^ | | | | - | | R | | S | | |  | |
| **96275** | Switzerland | Nyon-  Cheseaux Rd. | *T. aestivum*^b^ | | | | - | | R | | S | | |  | |

**^a^** infection type: R= resistant (IT=0-2); S= susceptible (IT=3-4); M= intermediate reaction (IT=2-3).

**^b^** approximation, i.e., the species powdery mildew was collected from is uncertain**.** In order to obtain a set of Swiss representative random mildew samples, a car was driven with the spore trap mounted on the roof in the main wheat growing areas of Switzerland. Those areas are dominated by bread wheat cultivars.
