# Supplementary material for: Reciprocal Hosts' Responses to Powdery Mildew Isolates Originating from Domesticated Wheats and Their Wild Progenitor
Source: Front Plant Sci. 2018 Feb 23;9:75. doi: 10.3389/fpls.2018.00075 (PMC5829517; doi:10.3389/fpls.2018.00075)
Supplement: Supplementary File — Statistical analyses. [file DataSheet1.DOC]

**Supplemental: Statistical** **analyses**

An unbalanced nested factorial model was employed for all three experiments with Y*_ijkl_* as the score of the l^th^ inoculated leaf segment of the j^th^ wheat line from the i^th^ species for each of the two *Bgt* isolates, by means of the formula:

**Y*_ijkl_* = µ + α*_i_* + β*_j_* + α*_i_*Xβ*_j_* + L_k_(β) + e*_ijkl_***

in which µ is the general mean, α_i_ is the wheat species factor with fixed effect [i = 1, 2, 3, or 4 for bread wheat, durum wheat, emmer wheat, or wild emmer, respectively]; β_j_ is the isolate factor with fixed effect, representing the two respective isolates that were assigned to each of the experiments (I, II, and III); α*_i_* × β*_j_* is the effect of the interaction between the i^th^ wheat species and the j^th^ isolate; L_l_(β) is the effect of the wheat line within a species (*k* = 1 to *n*); and *e_ijkl_* is the effect of the l^th^ inoculated leaf segment within the *k*^th^ wheat line in the *ij*^th^ α*_i_* X β*_j_* combination (*l* = 1 to *n*).
